# Approach to the diagnosis and management of suspected exercise-induced bronchoconstriction by primary care physicians

**DOI:** 10.1186/1471-2466-9-29

**Published:** 2009-06-15

**Authors:** James H Hull, Peter J Hull, Jonathan P Parsons, John W Dickinson, Les Ansley

**Affiliations:** 1 Faculty of Health and Social Care Sciences, Kingston University and St George's Hospital, London, UK; 2 Abbotsbury Road Primary Care Centre, Weymouth, Dorset, UK; 3 Division of Pulmonary, Allergy, Critical Care and Sleep Medicine, Ohio State University, Colombus, Ohio, USA; 4 Carnegie Centre for Performance and Wellbeing, Leeds Metropolitan University, Headingley, Leeds, UK; 5 School of Psychology & Sport Sciences, Northumbria University, Newcastle, UK

## Abstract

**Background:**

Exercise-related respiratory symptoms in the diagnosis of exercise-induced bronchoconstriction (EIB) have poor predictive value. The aim of this study was to evaluate how athletes presenting with these symptoms are diagnosed and managed in primary care.

**Methods:**

An electronic survey was distributed to a random selection of family practitioners in England. The survey was designed to assess the frequency with which family practitioners encounter adults with exercise-related respiratory symptoms and how they would approach diagnostic work-up and management. The survey also evaluated awareness of and access to diagnostic tests in this setting and general knowledge of prescribing asthma treatments to competitive athletes.

**Results:**

257 family practitioners completed the online survey. One-third of respondents indicated they encountered individuals with this problem at a frequency of more than one case per month. Over two-thirds of family practitioners chose investigation as an initial management strategy, while one-quarter would initiate treatment based on clinical information alone. PEFR pre- and post-exercise was the most commonly selected test for investigation (44%), followed by resting spirometry pre- and post-bronchodilator (35%). Short-acting β_2_-agonists were the most frequently selected choice of treatment indicated by respondents (90%).

**Conclusion:**

Family practitioners encounter individuals with exercise-related respiratory symptoms commonly and although objective testing is often employed in diagnostic work-up, the tests most frequently utilised are not the most accurate for diagnosis of EIB. This diagnostic approach may be dictated by the reported lack of access to more precise testing methods, or may reflect a lack of dissemination or awareness of current evidence. Overall the findings have implications both for the management and hence welfare of athletes presenting with this problem to family practitioners and also for the competitive athletes requiring therapeutic use exemption.

## Background

Exercise-induced bronchoconstriction (EIB) is highly prevalent in athletes at all levels of competition and its diagnosis and treatment is important to ensure their well-being [1,2]. Accurate diagnosis is essential to avoid misclassification and inappropriate treatment [3] and in competitive athletes is particularly important given potential implications on performance and strict regulations concerning the use of medications [4].

In January 2009, the World Anti-Doping Agency (WADA) implemented a number of significant changes in their policy for therapeutic use exemption (TUE) [5], the process through which an athlete may be permitted to use an otherwise prohibited substance to treat a medical condition. A key change to the policy concerns the TUE procedure for inhaled β_2_-agonists in asthma or EIB.

The change in the TUE policy will address an important aspect of EIB, namely the poor correlation between exercise-related respiratory symptoms and objective evidence of reversible airway narrowing [6,7]. It is currently recognized that clinical assessment alone is often insufficient to ensure a secure diagnosis of EIB; symptoms have poor predictive value and there exists a broad differential diagnosis [8]. Thus, the new policy requires submission of objective evidence of airway hyperresponsiveness to support diagnosis and as such has immediate implications for athletes but ultimately ramifications for the physicians overseeing their care.

In the UK, the majority of athletes with exercise-related respiratory symptoms will be assessed and treated in primary care. In the 12 months from 1^st ^August 2007 to 31^st ^July 2008 64% (n = 528) of all TUE applications for inhaled β_2_-agonists and corticosteroids were completed by family practitioners (personal communication, UK Sport). Of these applications less than 5% provided supporting evidence of reversible airway limitation, indicating that objective tests may not be commonly utilised in diagnosis. This assumption is in line with the observed practise of family practitioners in the US, where it has been argued that a lack of objective testing in diagnostic work-up may be leading to inaccurate or missed diagnoses [9].

Recent reviews highlight the difficulties faced by family practitioners when evaluating exercise-induced respiratory disorders [10], but problems may also arise because of an insufficient awareness or lack of access to appropriate tests. Currently however, exactly how family practitioners diagnose and manage athletes with suspected EIB remains undefined. We therefore conducted a survey of family practitioners in England designed to evaluate their clinical knowledge and approach to an athlete presenting with exercise-related respiratory symptoms suggestive of EIB.

## Methods

### Survey design and population

An electronic survey was designed to be distributed to family practitioners to explore the following aspects of diagnosis and management of exercise-related respiratory symptoms suggestive of EIB:

1) Frequency of consults of exercise-related respiratory symptoms in adults.

2) Strategies employed in diagnosis and management of a clinical scenario of suspected EIB.

3) Awareness of and access to tests used in diagnosis of EIB.

4) General knowledge of prescribing treatment for EIB in competitive athletes.

Data was collected over a six month period between February and July 2008. Surveys were anonomysed and processed by an independent analysis facility (Surveymonkey.com). The study was approved by the university research ethics committee (SUB_LA_1207) and all respondents were required to indicate informed consent before they were allowed to continue with the survey. Respondents were not compensated for completing the survey. Only fully completed surveys were used in final analysis.

### Survey instrument

The survey consisted of one clinical scenario (see below) and a series of multiple-choice questions. The scenario was based on a clinical presentation of an athlete with exercise-related respiratory symptoms encountered in primary care practice by one of the authors.

A 24-yr-old competitive cyclist consults you complaining of 'difficulty breathing' when exercising. He says symptoms start shortly after starting riding and reports difficulty 'catching his breath'. He also reports hearing occasional wheeze. He has no other medical history and otherwise feels very well. Examination and peak flow are normal. What is your next management step?

• *Advise reduction in exercise exposure*

• *Arrange further testing*

• *Prescribe treatment*

• *Reassure unlikely to be problem – no treatment or testing arranged*

• *Refer to specialist*

In order to simulate 'real-life' clinical practise and hence allow insight into investigation and management choices the questions within the survey had built-in logic steps i.e. successive questions were based on previous answers. This allowed an 'interactive' and hence more representative assessment of how family practitioners would approach the clinical scenario. It also permitted a further evaluation of management choice when respondents were provided with test results indicating no evidence of airway hyperresponsiveness (i.e. not fulfilling criteria to support a diagnosis of EIB) or when presented with a later re-consult with ongoing symptoms. The order in which the possible choices were presented was randomised for each respondent to avoid response bias. The survey did not permit respondents to revise their answers retrospectively.

Face validity was assessed in a pilot survey distributed to a cohort of twenty family practitioners with adjustments made prior to distribution of the final survey.

### Survey distribution

The survey was distributed as an electronic e-mail link, via primary care trusts (PCTs) to their e-mail distribution list of registered family practitioners/primary care practice managers. A random selection of representative PCTs in England were approached and 90% agreed to distribution. The nature of the distribution method did not allow an evaluation of successful delivery of e-mails and hence a determination of response rate. Furthermore the survey was only granted distribution on a single occasion within each PCT.

### Statistical analysis

Analysis of data is predominantly descriptive. Investigation of the influences of frequency of encounter (high (≥ 11 per year) and low (<11 per year)) and region (north, midlands, south) on responses were assessed using either Fisher's Exact Test (2 × 2) or Chi-squared Test for Independence. All percentages are rounded to the nearest integer. Significance was accepted at p < 0.05.

## Results

### Distribution and response rate

269 family practitioners started the survey and 257 (96%) completed the survey.

### Frequency of consult

**Figure 1:**
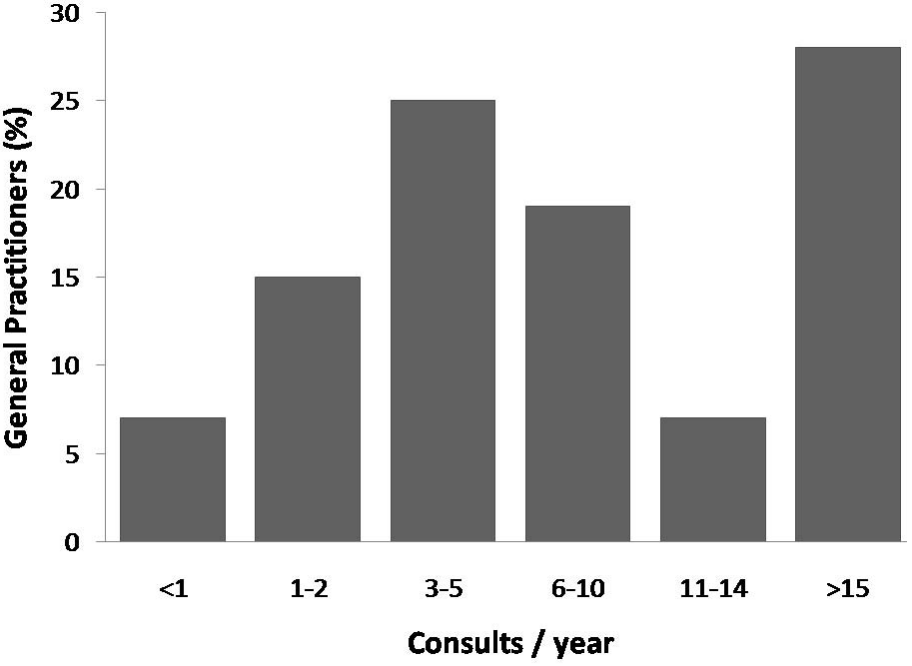
**Frequency of consult with exercise-related respiratory symptoms**.

Respondents' answers to the question '*Approximately how many patients a year do you see complaining of respiratory symptoms occurring during dedicated exercise?*' are shown in Figure 1. 35% of respondents indicated that they encountered at a frequency corresponding to at least one case per month. There were no regional differences in the frequency (i.e. high or low) of cases (p = 0.14).

### Strategies employed in diagnosis and management of clinical scenario

**Figure 2:**
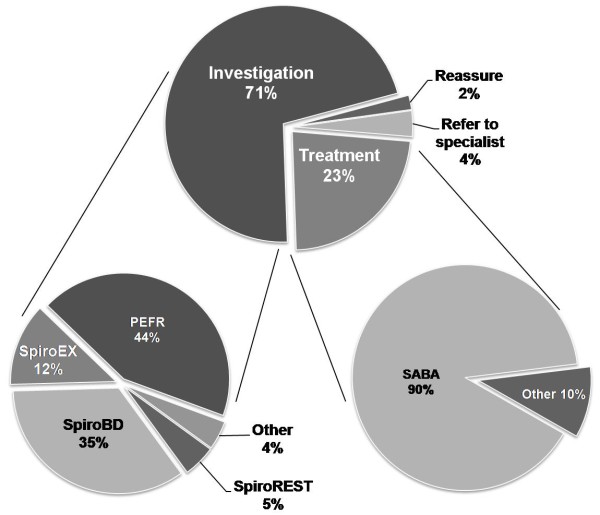
**Management strategy of clinical scenario**. *Definition of abbreviations*: **SpiroEX **= spirometry pre and post exercise; **SpiroBD **= spirometry pre and post bronchodilator; **SpiroREST **= spirometry at rest; **PEFR **= peak flow pre and post exercise; **SABA **= short-acting β_2_-agonist.

The management strategy selected by family practitioners when faced with the clinical scenario is illustrated in Figure 2. The choice of initial management strategy did not relate to frequency of encounter reported in the first question (p = 0.89).

### Choice of test

No respondents selected a bronchoprovocation test as an initial investigation. Choice of the three most commonly selected tests namely: PEFR; spirometry pre and post bronchodilator (spiroBD); and spirometry pre and post exercise (spiroEX), did not relate to consult frequency (p = 0.59) or geographical region (p = 0.45).

**Figure 3:**
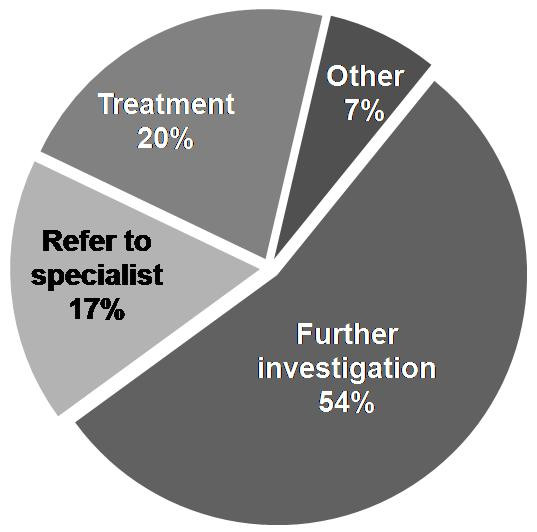
**Management strategy following an inconclusive initial test result (i.e. not supportive of EIB)**.

Respondents indicated they would select "other" investigations in 4% of cases. These included: a trial of inhaled β_2_-agonist during exercise, serial peak flow monitoring, peak flow with reversibility testing and in one case full blood count, chest radiograph, electrocardiogram and echocardiogram. The 'next-step' management strategy selected when respondents were presented with a test result that did not support diagnosis of EIB (e.g. *'there is less than 10% difference in PEFR before and after exercise'*) is shown in Figure 3.

### Choice of treatment

**Figure 4:**
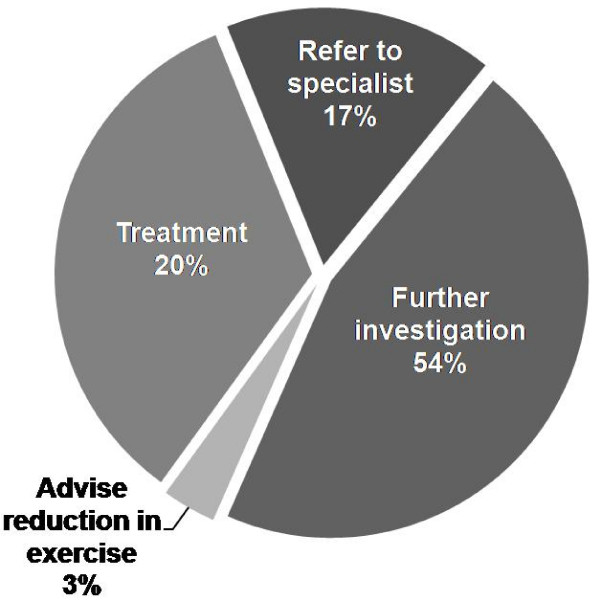
**Management strategy at re-consult following initial empirical treatment**.

90% of practitioners chose short-acting β_2_-agonists (SABA) as their initial treatment option and 2% selected to combine SABA with inhaled corticosteroids (ICS). In 3% of cases respondents indicated initial treatment with long-acting β_2_-agonists (LABA) without ICS if faced with this clinical scenario with 2% choosing LABA combined with ICS. A further 2% selected to treat with ICS on its own and 2% chose leukotriene receptor antagonists. No respondents selected oral corticosteroid, anti-cholinergics, sodium cromoglycate (cromones) or theophyllines (xanthines). Analysis was performed for those who selected empirical treatment as to management strategy at a re-consult two months later – '*The cyclist returns to see you 2 months later complaining of ongoing symptoms and feels that they are limiting his performance. What is your next management step*' (Figure 4). The instance of ICS treatment increased from 6% at initial consult to 75% at re-consult.

### Access to objective tests

In response to the question, '*To which of the following test(s), used in the diagnosis of exercise-induced bronchoconstriction, do you have access?*', 11% of the family practitioners indicated they have access to laboratory exercise testing while only 4% have access to eucapnic voluntary hyperpnea (EVH), metacholine or mannitol provocation testing. 85% of respondents had no access to any bronchoprovocation tests.

### General knowledge of prescribing treatment for EIB in competitive athletes

Responses to the question '*which of the following medications is a competitive cyclist permitted to use without notifying their governing body?*' are shown in Table 1. 8% of respondents thought that none of the medications were permitted for use without notification while 66% of respondents felt unsure.

**Table 1:** Responses to the question 'which of the following medications is a competitive cyclist permitted to use without notifying their governing body?

Medication	Responses (%)
Inhaled short-acting β_2_-agonists	20
Inhaled long-acting β_2_-agonists	14
Inhaled corticosteroid	9
Inhaled corticosteroid and short-acting β_2_-agonists	7
Inhaled corticosteroid and long-acting β_2_-agonists	6
Oral corticosteroid	1
Leukotriene receptor antagonists	18
Anticholinergics	11
Sodium cromoglycate	18
Theophyllines	4

## Discussion

The results from this survey indicate that it is common for family practitioners to encounter individuals with exercise-related respiratory symptoms; with over a third reporting at least one case per month. When faced with such a scenario nearly three quarters of respondents (71%) indicated they would select objective testing to diagnose EIB. However the most commonly selected tests, namely exercise PEFR (44%) and spirometry with bronchodilator (35%), have been found to have poor diagnostic accuracy for EIB [11,12]. In addition, a quarter of family practitioners (23%) indicated they would treat empirically based upon clinical features alone. Overall this raises concern that diagnosis of EIB may be inaccurate or indeed missed [6,7] and as such, these findings have implications for the welfare of athletes with this problem. They also have ramifications for competitive athletes given the mandatory requirement for objective evidence in application for inhaled β_2_-agonists TUE from Jan 2009; perhaps in particular for those athletes who may apply to renew their TUE on the back of an unsound initial diagnosis.

The International Olympic Committee-Medical Commission (IOC-MC) has recently renewed its consensus guidelines for the diagnosis of EIB in athletes with respiratory symptoms [13]. It is recommended that athletes with abnormal baseline spirometry (FEV_1 _< 80%, FEV_1_/FVC < 0.7) should be investigated initially with a bronchodilator challenge and otherwise with a bronchoprovocation challenge; the latter being defined as a test with the purpose of evaluating change in airway calibre in response to an airway challenge (e.g. exercise, EVH, methacholine, or mannitol provocation).

This guidance is based on the fact that bronchodilator testing in athletes is unlikely to detect airway reversibility in those with normal resting spirometry [1] and that bronchoprovocation testing has the highest sensitivity and specificity for diagnosis [14]. The IOC-MC guidelines also underline use of FEV_1 _as a marker of airway narrowing [8] given that use of PEFR may lead to misclassification [11] and as such is no longer recommended in guidelines or accepted by WADA. Please see relevant section in  for approach and algorithm recommended for UK athletes.

The choice of objective tests made by family practitioners when faced with this problem appears at odds with these recommendations. Bronchoprovocation was not selected by any respondent as a test of preference and PEFR was the most commonly used measure of airway narrowing. A key reason for this appears to be the limited access to bronchoprovocation challenges in primary care. In our cohort, 85% of family practitioners have no access to any sort of bronchoprovocation testing; 11% have access to laboratory-based exercise tests while only 4% reported access to EVH, methacholine or mannitol provocation testing. Our findings are supported by the UK TUE applications completed by family practitioners, which indicated PEFR in 28% of cases, spirometry in 3% and bronchoprovocation in 0.05% (personal communication, UK Sport). In contrast sports medicine specialists completing the TUE application provided supporting evidence of diagnosis with bronchoprovocation in 14% of cases. This may relate to differences in patient populations; however, does highlight the fact that the physicians most likely to initially encounter individuals with this condition have least access to the most accurate diagnostic tests. Furthermore, as of January 2009, the lack of access to these tests has important implications for the preparation of a medical file to fulfil the criteria for TUE and as such potentially limits the ability of family practitioners to manage competitive athletes with this problem.

The approach to an athlete with suspected EIB by family practitioners in England appears to contrast with that of family practitioners in the US [9]. On presentation of a similar case scenario, 81% of family practitioners in the US opted for empirical treatment and 18% for investigation vs. 23% and 72% respectively, in our study cohort. The practise of family practitioners in England appears more in line with US pulmonologists who were four-fold more likely than the family practitioners to employ testing initially. However, it should be noted that the US survey format only offered bronchoprovocation testing as means of investigation and in no instance was this method selected by responders in this study

When initiating treatment the vast majority of family practitioners in England (90%) indicated they would initiate treatment with a SABA alone. This is in line with guideline recommendations [12] and with reports of similar therapy preference in US family practitioners [9] and Finnish doctors [15]. In the treatment of EIB in athletes it is increasingly recognised that treatment with β_2_-agonists alone may not be adequate and has problems including tachyphylaxis and unfavourable side effects [16]. Furthermore, given the fact that there is recognised inflammatory component [17] and that athletes require medication regularly it has been recommended that early initiation of ICS is preferable [12]. In this study, 6% of family practitioners indicated they would initially treat with ICS, although this rose to 75% when faced with a re-consult at two months. Perhaps alarmingly, given the recommendations not to prescribe LABA without ICS, 3% of family practitioners chose this treatment strategy. Interestingly, despite an 'other' option being available in the answer section, no respondents indicated alternative recognized treatment options such as a warm-up [18], avoidance of triggers [2] or dietary modification [19,20]. Further work is needed to determine whether this may reflect a definitive choice on the part of family practitioners or be the result of a lack of dissemination or awareness of current evidence or teaching of sport and exercise medicine in England [21].

The approach chosen by many respondents to initiate treatment empirically is confounded by the poor correlation between subjective symptoms and objective evidence of airway narrowing [7]. It also presents a number of diagnostic difficulties if an individual represents with ongoing symptoms. The PRACTALL guidelines recommended that if EIB treatment is not successful then other diagnoses should be re-considered including vocal cord dysfunction, arterial hypoxemia and general poor physical fitness [10]. However, other possibilities include: insufficient treatment; poor therapy compliance; or ineffective inhaler technique. To explore this further we represented the athlete at two months after initiation of empirical treatment. Interestingly, almost half of respondents opted to arrange investigation at this point, whilst only one third opted to change treatment.

In treating competitive athletes, the majority of respondents (66%) indicated they were unsure which medication(s) a competitive athlete (in this scenario a cyclist) was permitted to use without notifying their governing body (Table 1). These findings are in keeping with previous surveys of family practitioners in the UK and France suggesting a limited knowledge of the implications of prescribing medication to this specialist population [22,23]. Although the onus remains on the athlete to inform a governing body of prohibited medication use, physicians should be aware of the process especially given the changes in the requirements for a medical file for TUE from January 2009.

Our study has a number of limitations. Firstly, similar to the report by Parsons and colleagues [9] the methods employed to distribute the survey meant that we were unable to accurately assess response rate. An electronically distributed method was selected in order to allow realistic feedback however as such did not allow us to determine delivery confirmation. To our knowledge, this study is the first to use this approach to survey family practitioners nationwide using the electronic e-mail database. We have no reason to believe bias in one direction within responses and furthermore our findings are supported by family practitioner completed TUE applications. Secondly, the wording of case scenario was selected to be suggestive of EIB, however it is acknowledged that the differential diagnosis is broad and potentially includes other respiratory and cardiac pathologies. We therefore provided an 'other' option and correspondingly a small proportion of family practitioners selected investigations, such as chest radiographs and electrocardiographs. Finally, the methods employed only permitted a single distribution of the survey. As such the findings would be supported and further validated by repeating assessment on an additional occasion.

## Conclusion

The findings from this study provide an insight into the manner in which athletes with exercise-related respiratory symptoms are diagnosed and managed in primary care. The results indicate that although some form of objective testing is often employed in diagnostic work-up, the tests most frequently employed are not the most accurate for the diagnosis of EIB.

Overall the findings have implications for the management and hence welfare of athletes presenting with exercise-related respiratory symptoms to primary care but also have important implications for competitive athletes requiring medical evidence in support of TUE application.

## Competing interests

JPP has received speaking fees from Glaxosmithkline inc., Merck inc and Schering-plough inc. Other authors have no competing interests.

## Authors' contributions

JHH made substantial contributions to conception and design, and the analysis and interpretation of data; was involved in drafting the manuscript and revising it critically for important intellectual content; and has given final approval of the version to be published. PJHmade substantial contributions to conception and design; was involved in revising the manuscript critically for important intellectual content; and has given final approval of the version to be published. JPPmade substantial contributions to the analysis and interpretation of data; was involved in revising the manuscript critically for important intellectual content; and has given final approval of the version to be published. JWDmade substantial contributions to the analysis and interpretation of data; was involved in revising the manuscript critically for important intellectual content; and has given final approval of the version to be published. LAmade substantial contributions to conception and design, and acquisition of data, and the analysis and interpretation of data; was involved in drafting the manuscript and revising it critically for important intellectual content; and has given final approval of the version to be published.

## Pre-publication history

The pre-publication history for this paper can be accessed here:


